# Identifying significant genes and functionally enriched pathways in familial hypercholesterolemia using integrated gene co-expression network analysis

**DOI:** 10.1016/j.sjbs.2022.02.002

**Published:** 2022-02-09

**Authors:** Zuhier Awan, Nuha Alrayes, Zeenath Khan, Majid Almansouri, Abdulhadi Ibrahim Hussain Bima, Haifa Almukadi, Hussam Ibrahim Kutbi, Preetha Jayasheela Shetty, Noor Ahmad Shaik, Babajan Banaganapalli

**Affiliations:** a Department of Clinical Biochemistry, Faculty of Medicine, King Abdulaziz University, Jeddah, Saudi Arabia; b Department of Genetics, Al Borg Diagnostics, Jeddah, Saudi Arabia; c Department of Medical Laboratory Sciences, Faculty of Applied Medical Sciences, King Abdulaziz University, Jeddah, Saudi Arabia; d Princess Al-Jawhara Center of Excellence in Research of Hereditary Disorders, King Abdulaziz University, Jeddah, Saudi Arabia; e Department of Science, Prince Sultan Military College of Health Sciences, Dhahran, Saudi Arabia; f Department of Pharmacology and Toxicology, Faculty of Pharmacy, King Abdulaziz University, Jeddah, Saudi Arabia; g Department of Pharmacy Practice, Faculty of Pharmacy, King Abdulaiziz University, Jeddah, Saudi Arabia; h Department of Biomedical Sciences, College of Medicine, Gulf Medical University, Ajman, United Arab Emirates; i Department of Genetic Medicine, Faculty of Medicine, King Abdulaziz University, Jeddah, Saudi Arabia; j Blockchain Applications in Healthcare Unit, Centre of Artificial Intelligence in Precision Medicine, KAU, Jeddah, Saudi Arabia

**Keywords:** Familial hypercholesterolemia, Gene expression, DEGs, PPI, Microarray, Network

## Abstract

Familial hypercholesterolemia (FH) is a monogenic lipid disorder which promotes atherosclerosis and cardiovascular diseases. Owing to the lack of sufficient published information, this study aims to identify the potential genetic biomarkers for FH by studying the global gene expression profile of blood cells. The microarray expression data of FH patients and controls was analyzed by different computational biology methods like differential expression analysis, protein network mapping, hub gene identification, functional enrichment of biological pathways, and immune cell restriction analysis. Our results showed the dysregulated expression of 115 genes connected to lipid homeostasis, immune responses, cell adhesion molecules, canonical Wnt signaling, mucin type O-glycan biosynthesis pathways in FH patients. The findings from expanded protein interaction network construction with known FH genes and subsequent Gene Ontology (GO) annotations have also supported the above findings, in addition to identifying the involvement of dysregulated thyroid hormone and ErbB signaling pathways in FH patients. The genes like *CSNK1A1, JAK3, PLCG2, RALA, and ZEB2* were found to be enriched under all GO annotation categories. The subsequent phenotype ontology results have revealed *JAK3I*, *PLCG2, and ZEB2* as key hub genes contributing to the inflammation underlying cardiovascular and immune response related phenotypes. Immune cell restriction findings show that above three genes are highly expressed by T-follicular helper CD4^+^ T cells, naïve B cells, and monocytes, respectively. These findings not only provide a theoretical basis to understand the role of immune dysregulations underlying the atherosclerosis among FH patients but may also pave the way to develop genomic medicine for cardiovascular diseases.

## 1 Introduction

FH is a metabolic condition where defective uptake of circulating LDL particles by the liver leads to the elevation of serum cholesterol levels (Awan et al., 2021). This chronic condition causes a high cholesterol deposition in the inner walls of arteries, hastening atherosclerosis and increase the susceptibility to develop premature cardiovascular diseases (CVD) (Alhabib et al., 2021, Fantus et al., 2013). The estimated risk of premature CVDs is 20-fold higher for FH patients, compared to the general population (Villa et al., 2017). FH displays genetic heterogeneity. The disease can be either monogenic or polygenic, with a variety of molecular etiologies. Up to 80% of the FH patients have heterozygous loss-of-function (LoF) mutations in the *LDLR* (Berberich and Hegele, 2019); while a minority have LoF mutations in the receptor-binding functional segments of *APOB* (Andersen et al., 2016); or gain-of-function (GoF) mutations in *PCSK9* (Abifadel et al., 2003). Biallelic *LDLRAP1* gene mutations also exist, but to a much lesser extent.

The monogenic forms of FH can be classified as either homozygous FH (HoFH) or heterozygous FH (HeFH) based on the allelic status of *LDLR* (Warden et al., (MA)2000.). In the majority of the investigated populations, HoFH is seen one in one hundred sixty thousand to three hundred thousand persons, while HeFH is seen one in two hundred fifty to three hundred persons (Berberich and Hegele, 2019). In comparison to HeFH, HoFH results in a much more severe disease manifestation. The latter also increases the rate of surgical intervention and death in the mid-twenties (Raal and Santos, 2012). Approximately, 30–70% of the clinically diagnosed FH patients, do not carry pathogenic *PCKS9, LDLR*, or *APOB* variants. However, some of them may carry rare frequency LoF variants in other FH genes like *LIPA, ABCG8, APOE or ABCG5*. Additionally, several high frequency allelic variants, which together act to influence serum LDL-C concentrations, are also reported (Paththinige et al., 2017).

FH is traditionally studied as a classical Mendelian disease, where causative variants perturb LDL binding, internalization and transportation mechanisms, resulting in the elevation of cholesterol laden LDL levels in the serum (Brænne et al., 2014, Wu et al., 2014, Marks et al., 2003). However, the effects of individual variants and the functional interactions between different variants in the protein is likely to be influenced by the expression status of that protein (Li et al., 2019). So, there is a greater need to study the gene expression changes in FH patients, which potentially contributes to atherosclerosis (Soutar and Naoumova, 2007). In this context, studying high-throughput gene expression profiling technologies like cDNA microarrays can provide a rapid and quantifiable assessment of thousands of genes spanning the whole genome (Qin et al., 2021). Moreover, unprecedented developments taking place in statistical modelling and bioinformatics approaches, over the past few decades, have given us a greater advantage in investigating the impact of gene expression changes on protein networks and pathways (Sabir et al., 2019, Sabir et al., 2020, Banaganapalli et al., 2021, Banaganapalli et al., 2020). Additionally, they have also assisted in the identification of potential druggable biomarkers (Huang, 1999).

The new bioinformatic methods like CIBERSORT, TIMER and EPIC can effectively characterize immune cell composition of different diseases using large-scale gene expression data (Craven et al., 2021, Xie et al., 2021). Therefore, owing to the lack of sufficient information, this study aims to uncover potential genetic biomarkers for FH. A broad range of advanced computational methods, including differential gene expression analysis, protein network mapping, hub gene identification, functional enrichment of biological pathways, and immune cell enrichment analysis were used to get a deeper understanding about the molecular abnormalities, which contributes to the development of FH and the associated health complications.

## 2 Materials and methods

### 2.1 FH transcriptome datasets

Two FH transcriptomic datasets were taken from Array Express (Athar et al., 2019). The first dataset (E-GEOD-13985) consists of the white blood cell expression profiles of five FH patient samples and five controls (sex, age, body mass index and life style habits matched participants), generated on the Affymetrix GeneChip U133 + 2 (Human Genome) (Režen et al., 2019). The second expression dataset (E-GEOD-6088) included the white blood cell expression profiles of 10 FH patients and 13 controls (sex, age, body mass index and life style habits matched participants), generated via the HG-U133 + 2 chip (Mosig et al., 2008). The complete details of the study layout, samples used, RNA extraction, and array hybridization protocols are discussed in the original publication (Mosig et al., 2008).

### 2.2 Raw expression signals, pre-processing, and detection of DEGs

The processing of raw expression signals was performed with the Bioconductor package in R software program (Irizarry et al., 2003, Gautier et al., 2004). The noise reduction and standardization of the sample data were achieved by uploading .CEL files into the Bioconductor tool affy program. The unprocessed expression signals were standardized to median values by applying the Robust Multiarray Average (RMA) method (Ritchie et al., 2007). The genes showing 1.5-fold change (FC) expression difference and which cleared Benjamini and Hochberg’s false discovery rate (FDR) with p-value ≤ 0.05 were flagged as DEGs (Gentleman et al., 2004). The volcano and mean plots were generated using R Program limma package. All the expression probes were referenced against Entrez gene IDs, and duplicate transcripts were removed. The DEGs common to both the datasets were identified with Venny 2.1 webtool (www.bioinfogp.cnb.csic.es/tools/venny).

### 2.3 Network construction and GO-Annotations

The STRING webserver helps in studying the direct or indirect interactions between expressed proteins (Banaganapalli et al., 2020). In this context, the DEGs obtained from the above steps were used to construct the protein–protein interaction (PPI) network with help of STRING database (Szklarczyk et al., 2017). The maximum enrichment p-value and the minimal average local clustering coefficient values of the PPI networks were < 1.0x10^-16^ and > 0.4, respectively. The confidence score of > 0.4 and the maximum additional interactions of 10 nodes were used to build the network. Then GO annotation analysis was performed to find the most significant terms using the Enrichment Analysis Visualization Appyter (Clarke et al., 2021). This Appyter allows the generation of scatterplots, bar charts, hexagonal canvas, Manhattan plots, and volcano plots (https://appyters.maayanlab.cloud/#/Enrichment_Analysis_Visualizer). All the protein–protein network was illustrated using Cytoscape3.8.2 (Shannon et al., 2003).

### 2.4 Identification of network clusters

A Cytoscape plugin called as Molecular Complex Detection (MCODE), was utilized to look for clusters within the PPI networks using the default settings like, k‐core is equal to 2, node score of 0.2, degree cutoff of 2, and network depth of 100 (Bader and Hogue, 2003). Then, hub genes were identified based on the high MCODE scores (>5) using the cytoHubba plug-in of Cytoscape. Furthermore, subnetworks were created using known primary and secondary FH candidate genes (*LDLR, LDLRAP1, PCSK9, APOB, APOA2, GHR, ABCA1, SMARCA4, and EPHX2*) and hub genes.

### 2.5 Enrichr-GO annotations of cluster networks

The PPI subnetworks created with hub genes and FH candidate genes were provided as an input to the Enrichr webserver (https://maayanlab.cloud/Enrichr) (Xie et al., 2021). The GO analysis option available in Enrichr provides biological information regarding the involvement of PPI subnetworks in Biological-Processes (BPs), Cellular-Components (CC), Molecular-Functions (MFs) and pathway database (Kyoto-Encyclopedia of Genes and Genomes Pathways (KEGG)). The Benjamini-Hochberg (BH) step-down method was used for statistical assessment of GO terms at a *p*-value of < 0.05 and a combined enrichment score of > 20. The REVIGO tool was then used to summarize GO terms and to remove redundant terms (Supek et al., 2011).

### 2.6 Open target phenotype identification

The hub genes selected from GO enrichment network findings were further explored in the Open Targets Platform. (http://platform.opentargets.org). This webserver provides unified access to diverse computational tools to query the causality relationship, including physical binary interactions, enzymatic reactions, or functional relationships between therapeutic targets and disease phenotypes. The query gene list was provided as an input option in the search box and the output was in the form of an evidence score for a given target-disease pair. The open target platform association score at a < 0.5 cutoff value was considered significant to detect the expression status of druggable molecular targets.

### 2.7 Mapping immune cell specific transcriptional signatures

The DICE database was used to understand how key genes enriched in all GO annotation terms show immune cell specific variations (https://dice-database.org/genes/). This database provides comprehensive information on immune cell expression. Upon providing the query gene list, this tool displays the expression level of genes in transcripts per million (TPM) on the x-axis, and cell types are sorted based on the y-axis of box plot graphs. The DEGs across all the cell types were identified by the DESeq package. Here, cell sorting is performed in reference to the gene expression values, arranged from the highest one to the lowest one. The x- and y-axes show pair-wise comparisons of two cell types. Statistical significance from log2 fold changes to P values is demonstrated in an interactive manner.

## 3 Gene-Drug interactions

The druggability of the query genes was determined using the Drug–Gene Interaction database (DGIdb) (Cotto et al., 2018). DGIdb is a centralized repository for data on drug-gene interactions and the druggability of each query gene is collected from multiple sources. The default filters like approved drugs, cytotoxic agents, and immunotherapeutic drug interactions from nine disease-agnostic source databases were used during the searching step. The drug-gene interaction output data of queried genes was further filtered based on interaction types (activator or inhibitor) and molecular category of gene (transporter, domain, surface protein, G-protein receptor etc.), at a cut-off interaction score of > 0.03.

## 4 Results

### 4.1 The analysis of DEGs

**Fig. 1:**
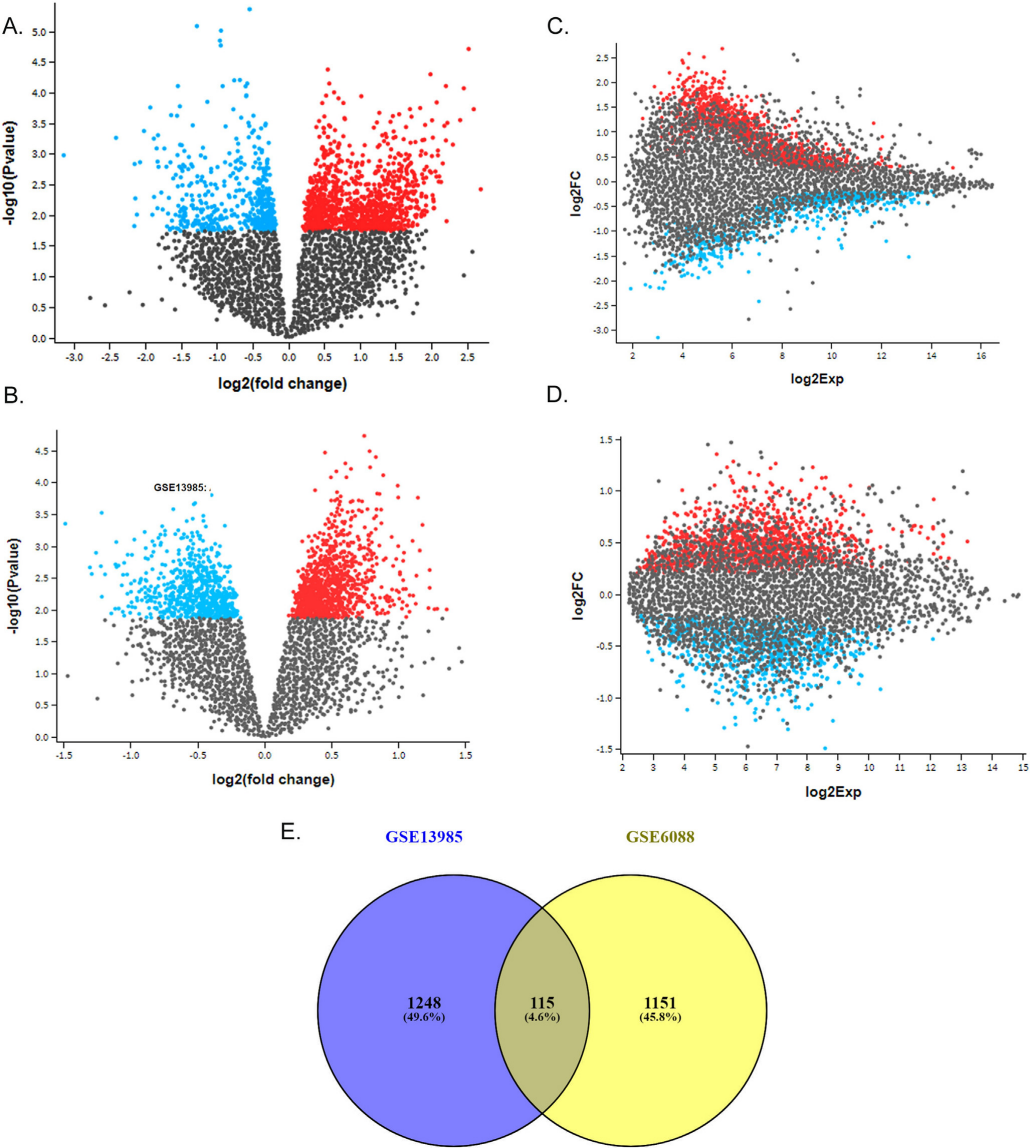
**A & B** represents the volcano plots built based on their gene expression distribution across the different datasets. **C&D** shows the mean difference (MD) plots, which represents log2 fold change (FC) verses average log2 expression values. All the up and down regulated genes are shown in red and blue dots, respectively. **E** shows venny diagram representing the shared DEGs between the two expression datasets (GSE13985 and GSE6088).

The gene expression profile of E-GEOD-13985 dataset revealed a total of 1363 DEGs (650 upregulated and 713 downregulated). In E-GEOD-6088 dataset, there were 1266 DEGs (589 up-regulated and 677 down-regulated). The comparison of DEGs across the datasets using VENN plot, has detected 115 shared genes (47 upregulated and 68 down regulated) (Fig. 1**A-E**). These shared genes were selected for further functional analysis with an underlying assumption that they have a critical role in hypercholesterolemia (Table 1).

**Table 1:** The top ten DEGs (FC > 1.5) of two FH datasets (GSE6088 and GSE13985).

**Dataset**	**ID**	**adj.P.Val**	**P.Value**	**t**	**B**	**logFC**	**Gene.symbol**
GSE6088	224590_at	0.7801	0.1398758	1.529775	−4.54401	3.26	*XIST*
	244635_s_at	0.3488	0.0005374	4.026184	−0.40308	2.39	*SH3BGRL2*
	230170_at	0.3488	0.0004439	4.10344	−0.26005	2.33	*OSM*
	1568751_at	0.5388	0.0057808	3.046385	−2.19903	2.16	*RGS13*
	229823_at	0.0684	6.26E-06	−5.83931	2.81582	−2.58	*RIMS2*
	206700_s_at	0.6963	0.0550873	−2.02216	−3.88611	−2.73	*KDM5D*
	211571_s_at	0.6655	0.0305771	−2.30615	−3.45333	−2.55	*VCAN*
	201909_at	0.8386	0.2596399	−1.15613	−4.94637	−2.23	*RPS4Y1*
GSE13985	222413_s_at	0.337	0.0004397	4.839746	0.00587	1.490431	*KMT2C*
	204141_at	0.795	0.2467529	1.219486	−5.02419	1.250299	*TUBB2A*
	213674_x_at	0.389	0.0112819	3.005055	−2.58596	1.145583	*IGHD*
	227510_x_at	0.424	0.0174138	2.768416	−2.94226	1.12653	*MALAT1*
	202110_at	0.402	0.0135719	−2.90435	−2.73765	−1.32733	*COX7B*
	209160_at	0.374	0.0097469	−3.08477	−2.46598	−1.35891	*AKR1C3*
	206698_at	0.625	0.0832517	−1.89427	−4.20908	−1.3766	*XK*
	232535_at	0.514	0.0395612	−2.31695	−3.61287	−1.45199	*RSBN1L*

### 4.2 Analysis of STRING PPI network and Appyters-GO annotations

**Fig. 2:**
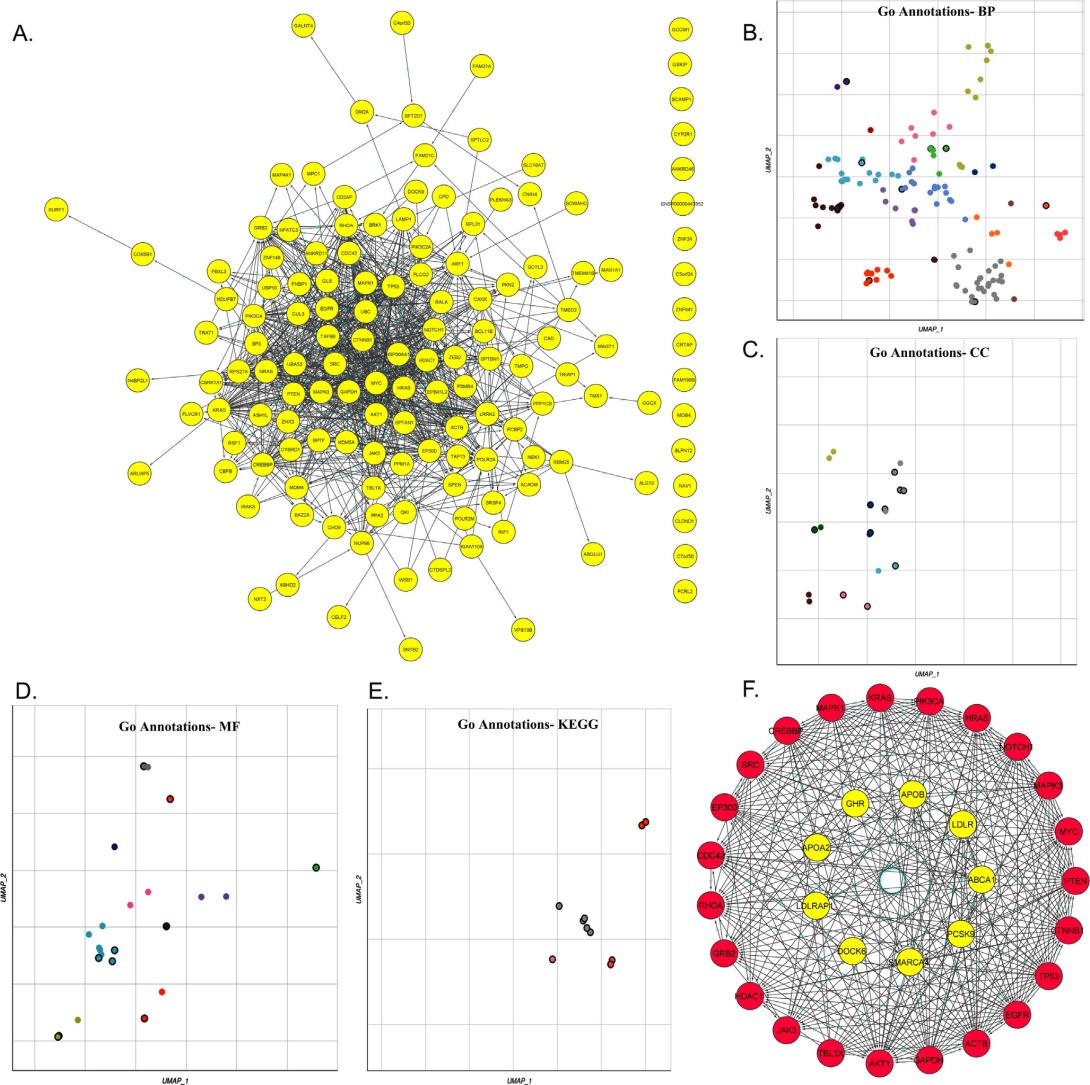
**A.** The PPI network showing interactions between FH genes through nodes and edges. **B-E** The scatterplots showing functional enrichment of DEGs against different GO-terms like biological processes (BP), molecular function (MF), and cellular components (CC) based their p-values. F. PPI network showing interactions between hub genes (red) interacting with known FH genes (in yellow).

PPI networks demonstrate the physical connectivity among different protein partners, and their disturbance could negatively impact broad range of molecular mechanisms essential for the cellular function. The PPI networks of 115 shared DEGs were expanded by 10 additional interactor proteins with a confidence value of > 0.4. The protein–protein interaction network generated was characterized with 151 nodesconnected to 323 edges with 4.28 Å as the average node degree and avg, local clustering-coefficient value of 0.473 (Fig. 2**A**).

GO annotations provide biological knowledge about genes and their protein products. GO enrichment findings of 115 shared DEGs with Appyter tool, has shown their annotations in 153 BP, 22 CC, 29 MF, and 10 KEGG pathways. The top key enrichment terms were, the controlling the Wnt signaling pathway (GO:00060828) under BP category with an p-value of < 0.0001, coated vesicle (GO:0030135) under cellular component category with an p-value of < 0.001, pyruvate transmembrane transporter activity (GO:0050833) under molecular functions category with an p-value of < 0.0003 and mucin type O-glycan biosynthesis pathway (map00512) under pathways term (p-value of < 0.01) (Fig. 2**B-E,** Table 2).

**Table 2:** Top 4 gene ontology enrichment terms obtained by Appyters-GO annotations for biological processes (BP), cellular components (CC), molecular functions (MF), and Kyoto Encyclopedia of Genes and Genomes (KEGG) pathways.

**Go Process**	**term**	**p-value**	**q-value**
**BP**	Regulation of canonical Wnt signaling pathway (GO:0060828)	0.000137	0.063634
	Regulation of mRNA splicing, via spliceosome (GO:0048024)	0.000201	0.063634
	Anion homeostasis (GO:0055081)	0.000347	0.063634
	Regulation of dendritic cell cytokine production (GO:0002730)	0.000347	0.063634
**CC**	Coated vesicle (GO:0030135)	0.001593	0.183228
	Transcription factor TFIID complex (GO:0005669)	0.014586	0.244397
	Intracellular membrane-bounded organelle (GO:0043231)	0.014996	0.244397
	Endoplasmic reticulum membrane (GO:0005789)	0.026371	0.244397
**MF**	Pyruvate transmembrane transporter activity (GO:0050833)	0.000347	0.052392
	Cadherin binding (GO:0045296)	0.003165	0.18173
	mRNA binding (GO:0003729)	0.00492	0.18173
	Oxidoreduction-driven active transmembrane transporter activity (GO:0015453)	0.004995	0.18173
**KEGG**	Mucin type O-glycan biosynthesis	0.019385	0.620672
	Alanine, aspartate and glutamate metabolism	0.020414	0.620672
	Various types of N-glycan biosynthesis	0.022539	0.620672
	D-Glutamine and D-glutamate metabolism	0.029401	0.620672

### 4.3 FH network clusters and Enrichr-GO annotations

In protein networks, protein partners in a cluster share similar functional characteristics, and hub genes are the biologically interrelated nodes in cluster. The MCODE plugin of the Cytoscape identifies the clusters in a given protein network. The protein interaction network of DEGs revealed a cluster with 33 nodes and 429 edges with an Maximum Clique Centrality (MCC) algorithm score of 26.812. This MCODE cluster was then enriched with 9 FH candidate genes (*APOA2, APOB, GHR, LDLR, LDLRAP1, ABCA1, PCSK9, SMARCA4,* and *EPHX2*) to extend the PPI network for subsequent analysis (Fig. 2**F**). The FH nodes interacting with DEG edges with an average STRINGDB interaction evidence score of >0.5 were filtered out. Of the FH genes, *APOA2* is found interacting with 6 genes (*APOB, ABCA1, TBL1X, CREBBP, EP300, and LDLR*), *APOB* interacting with 8 genes (*PCSK9, ABCA1, LDLR, LDLRAP1, ACTB, GAPDH, AKT1, and APOA2*), GHR interacting with 7 genes (*TP53, GRB2, JAK3, SRC, MAPK1, MAPK3,* and *PIK3CA*), *LDLR* interacting with 10 genes (*LDLRAP1, GAPDH, ACTB, AKT1, EGFR, CTNNB1, ABCA1, PCSK9, APO2A*, and *APOB*), *LDLRAP1* interacting with 3 genes (PCSK9, APOB, and LDLR), *ABCA1* interacting with 12 genes (*LDLR, PCSK9, CREBBP, EP300, CDC42, APOPA2, APOB, AKT1, TBL1X, GAPDH, ACTB*, and *RHGA*), PCSK9 interacting with 14 genes (*APOB, KRAS, HRAS, CDC42, AKT1, GAPDH, LDLRAP1, LDLR, ACTB, PTEN, MAPK3, ABCA1, TP53*, and *EGFR), SMARCA4* interacting with 15 genes (*EP300, KRAS, HRAS, PIK3CA, NOTCH1, CREBBP, PTEN, AKT1, TP53, HDAC1, GAPDH, EGFR, ACTB, CTNNB*, and *MYC*).

**Fig. 3:**
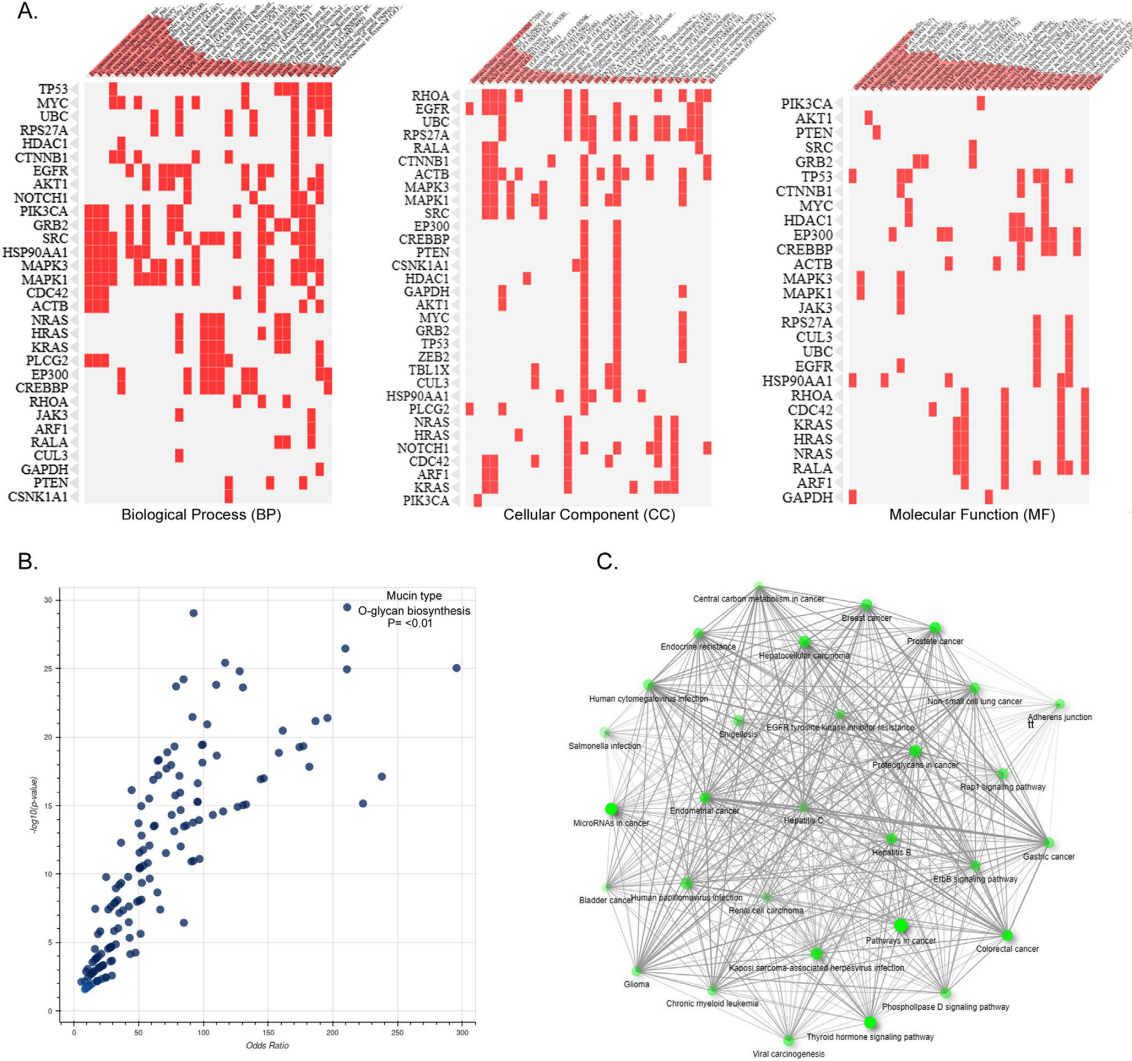
A. The Clustergram of Enrich tool shows the enrichment of FH genes under different GO terms like biological processes (BP), molecular function (MF), and cellular components (CC). The input genes are represented in columns and matrices represents the enriched terms of those genes. B. The volcano plots illustrating the significance of gene sets are represented by each dot against the odds ratios. x- and y- axes shows odds ratios and -log(p-value) of the each gene set, respectively. C. The network wiring shows interconnectivity between different pathways enriched by hub genes.

**Fig. 4:**
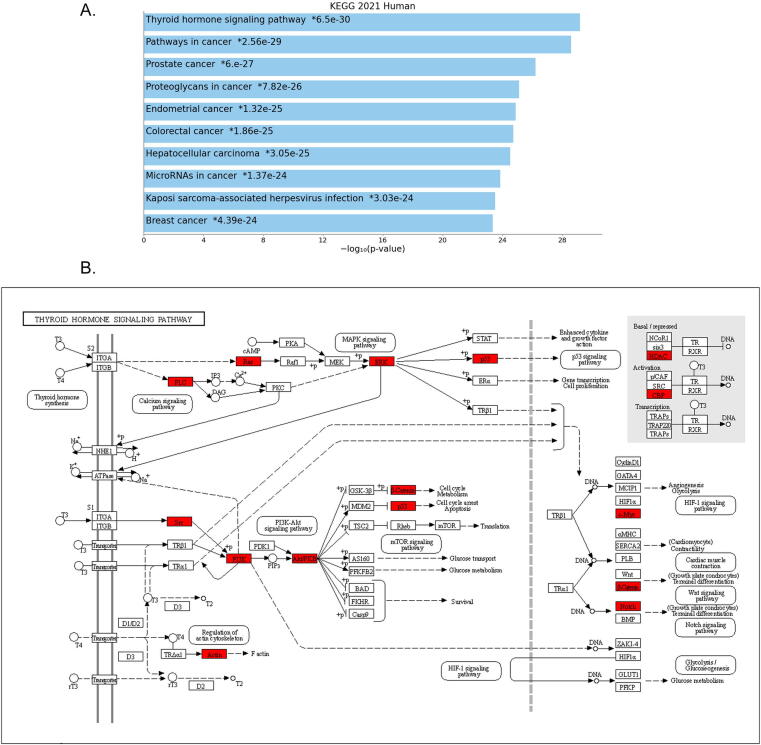
A. Bar graph represents the significant pathways enriched by DEGs at a p-value of < 0.05. B. The thyroid hormone signaling pathway with red color boxes highlighting the hub genes.

We used Enrichr-GO enrichment analysis to further understand how cluster genes in the network are perturbed in the FH condition. Their contribution in 475 BP, 49 CC, 92 MF, and 31 KEGG pathways was discovered. (Fig. 3**A-C**). The most significant term in the biological processes category was the regulation of intracellular signal transduction (GO:1902531) with an p-value of 6X10^-11^, followed by positive regulation of transcription (GO:00045893) with an p value of 1.31E^-10^. Under the cellular component category, the most significant term was the intracellular membrane-bounded organelle (GO:0043231) with a p-value of 2.51X10^-08^, followed by the cell-substrate junction (GO:0030055) with a p-value of 1.98X10^-11^. Under the molecular functions category, RNA pol II-specific DNA-binding-TF-binding (GO:00061629) with a p-value of 6.08X10^-07^, followed by DNA-binding TF binding (GO:00140297) with a p-value of 1.03X10^-06^ were the most significant terms. In KEGG pathways category, thyroid hormone signaling pathway was the key enrichment term with a P-value of 2.75X10^-26^ followed by the ErbB signaling pathway with a P-value of 2.06X10^-18^. Of all the genes, *CSNK1A1, JAK3, PLCG2, RALA*, and *ZEB2* were found to be enriched under all GO annotation categories (Fig. 4**A-B,** Table 3).

**Table 3:** Top 4 GO terms after cluster enrichment with the 9 FH genes obtained by Enrichr-GO annotations for biological processes (BP), cellular components (CC), molecular functions (MF), and Kyoto Encyclopedia of Genes and Genomes (KEGG) pathways.

**Annotation**	**Term**	**Overlap**	**P-value**	**Adjusted P-value**	**Odds Ratio**	**Combined Score**	**Genes**
BP	MAPK cascade (GO:0000165)	13/303	7.48E-16	9.76E-13	44.10362069	1536.088384	*CUL3; EGFR; NRAS; PIK3CA; MYC; UBC; MAPK1; GRB2; KRAS; RPS27A; HRAS; JAK3; MAPK3*
	Fc-gamma receptor signaling pathway involved in phagocytosis (GO:0038096)	9/71	1.91E-15	9.76E-13	120.3931452	4080.57903	*CDC42; HSP90AA1; PIK3CA; SRC; PLCG2; MAPK1; GRB2; ACTB; MAPK3*
	Fc-gamma receptor signaling pathway (GO:0038094)	9/72	2.18E-15	9.76E-13	118.4761905	3999.913746	*CDC42; HSP90AA1; PIK3CA; SRC; PLCG2; MAPK1; GRB2; ACTB; MAPK3*
	Fc receptor mediated stimulatory signaling pathway (GO:0002431)	9/74	2.82E-15	9.76E-13	114.8192308	3846.700768	*CDC42; HSP90AA1; PIK3CA; SRC; PLCG2; MAPK1; GRB2; ACTB; MAPK3*
CC	Focal adhesion (GO:0005925)	11/387	1.63E-11	8.61E-10	26.0518617	647.0765271	*CDC42; RALA; ARF1; SRC; MAPK1; CTNNB1; KRAS; EGFR; ACTB; RHOA; MAPK3*
	Cell-substrate junction (GO:0030055)	11/394	1.98E-11	8.61E-10	25.56657963	630.099147	*CDC42; RALA; ARF1; SRC; MAPK1; CTNNB1; KRAS; EGFR; ACTB; RHOA; MAPK3*
	Vesicle (GO:0031982)	8/226	2.56E-09	7.41E-08	28.9893578	573.5459587	*UBC; PLCG2; AKT1; RPS27A; GAPDH; EGFR; ACTB; RHOA*
	Intracellular membrane-bounded organelle (GO:0043231)	24/5192	2.51E-08	5.45E-07	7.63622291	133.6477075	*CREBBP; HSP90AA1; NOTCH1; HDAC1; CSNK1A1; CUL3; PTEN; EGFR; ACTB; RHOA; ZEB2; MYC; UBC; PLCG2; MAPK1; EP300; AKT1; CTNNB1; GRB2; TBL1X; RPS27A; GAPDH; TP53; MAPK3*
MF	GTP binding (GO:0005525)	7/189	2.09E-08	1.31E-06	29.26775148	517.5648946	*CDC42; RALA; ARF1; NRAS; KRAS; HRAS; RHOA*
	Phosphatase binding (GO:0019902)	6/114	2.93E-08	1.31E-06	40.86213992	708.7297409	*MAPK1; CTNNB1; JAK3; TP53; EGFR; MAPK3*
	Purine ribonucleoside triphosphate binding (GO:0035639)	9/460	3.93E-08	1.31E-06	16.22727273	276.6892164	*CDC42; RALA; ARF1; NRAS; HSP90AA1; AKT1; KRAS; HRAS; RHOA*
	Kinase binding (GO:0019900)	9/461	4.01E-08	1.31E-06	16.19054204	275.7614857	*CDC42; SRC; PLCG2; CTNNB1; GRB2; TP53; EGFR; ACTB; RHOA*

### 4.4 Open target phenotype identification

**Fig. 5:**
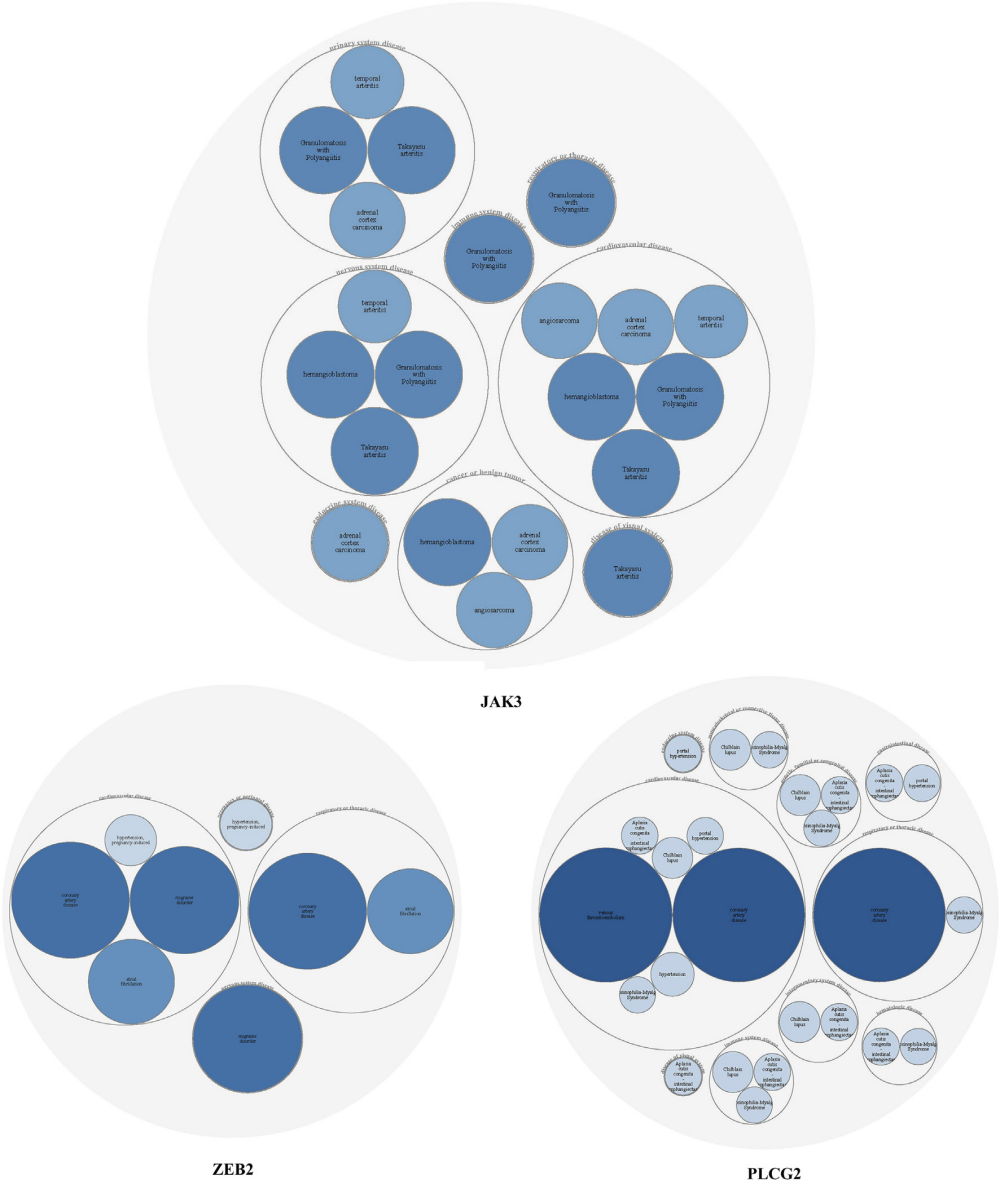
Open Target Platform based gene-disease phenotype analysis showing the association of *JAK3, ZEB2* and *PLCG2* genes with cardiac and immune related diseases. The disease names are mentioned in bubble, the shade and size of bubble color determine based on the gene-disease assocation score of > 0.1.

The open target platform makes use of the systematic “experimental factor ontology” (EPO) to identify both direct and indirect correlations between the target gene and disease phenotype. In this context, we explored the association of the above mentioned *CSNK1A1, JAK3, PLCG2, RALA*, and *ZEB2* genes sourced in cardiac and immune disorder-related diseases in the Open Target Platform. Of those five genes, three genes *(JAK3, PLCG2, and ZEB2*) have shown a significant overall association score of 0.37 in both cardiac and immune disorder-related diseases. *ZEB2* and *PLCG2* genes have shown stronger associations with coronary artery disease, with an association value>0.3. *JAK3* deficiency is significantly correlated with a multitude of immune-associated disorders, includes tuberculosis, severe combination immunodeficiency, and rheumatoid arthritis, with a score>0.5 (Fig. 5**,** Table 4).

**Table 4:** Top 5 Open Target associated disease phenotypes for the 3 genes that were found common in all Enrichr-GO annotation terms (JAK3, ZEB2, and PLCG2).

**Gene**	**name**	**Overall Association Score**	**Genetic Associations**	**Somatic Mutations**	**drugs**	**Pathways Systems Biology**	**textMining**	**Rna Expression**	**Animal Models**
*JAK3*	T-B + severe combined immunodeficiency due to *JAK3* deficiency	0.79354891	0.86210536	No data	No data	No data	No data	No data	0.639792477
	Rheumatoid arthritis	0.60889198	No data	No data	0.978608	No data	0.459466938	No data	No data
	Ulcerative colitis	0.58868596	No data	0.455948098	0.845344	No data	0.094824248	0.105747791	0.311929292
	Ankylosing spondylitis	0.47300062	No data	No data	0.777442	No data	0.012158616	No data	No data
	Immune system disease	0.4638375	No data	No data	0.759913	No data	0.061279424	No data	No data
*ZEB2*	Coronary artery disease	0.37565129	0.61093509	No data	No data	No data	0.139655214	No data	No data
	Migraine disorder	0.32028527	0.52684495	No data	No data	No data	No data	No data	No data
	Crohn's disease	0.28942152	0.47607643	No data	No data	No data	No data	No data	No data
	Inflammatory bowel disease	0.27025168	0.4427197	No data	No data	No data	0.036475848	No data	No data
	Acute myeloid leukemia	0.25080082	No data	No data	No data	0.371977657	0.811412861	No data	No data
*PLCG2*	*PLCG2-*associated antibody deficiency and immune dysregulation	0.69828022	0.78440264	No data	No data	No data	0.100353358	No data	No data
	*NLRP12-*associated hereditary periodic fever syndrome	0.56458052	0.68230439	No data	No data	No data	No data	No data	No data
	Venous thromboembolism	0.4358949	0.71701402	No data	No data	No data	No data	No data	No data
	Coronary artery disease	0.4320773	0.71073435	No data	No data	No data	No data	No data	No data
	Inflammatory bowel disease	0.40521025	0.6638044	No data	No data	No data	0.054713772	No data	No data

### 4.5 Immune cells expression analysis

**Fig. 6:**
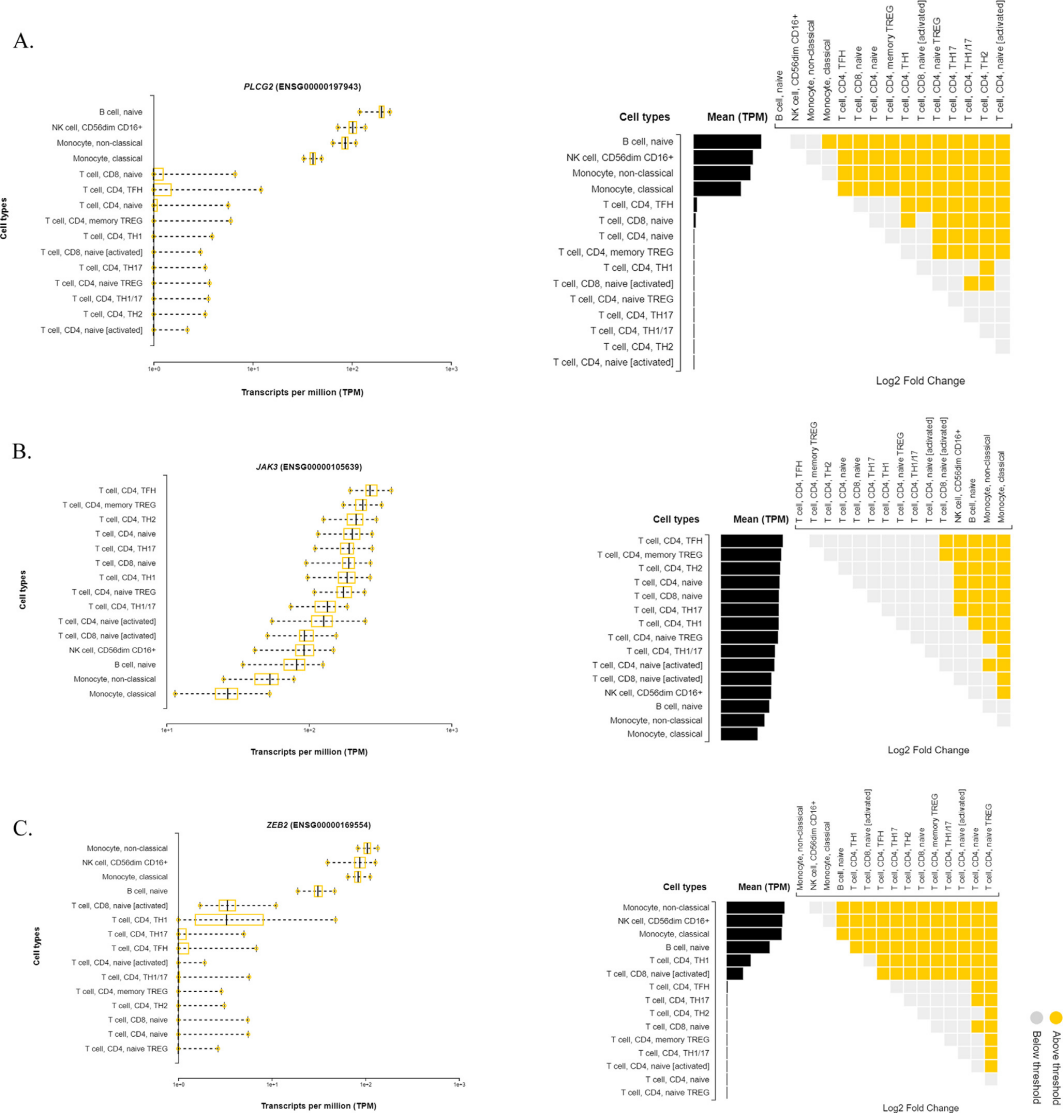
DICE tool shows the expression restriction of (A) *JAK3,* (B) *ZEB2* and (C) *PLCG2* genes across 13 immune cells in the form of box plots and pairwise comaprsion graphs.

The transcript expression analysis of *JAK3, PLCG2*, and *ZEB2* genes has been performed to explore their immune cell type restriction. The *JAK3* is expressed by T-follicular helper CD4^+^ T cells (272 TPM), memory TREGs CD4^+^ T cell (235 TPM), TH2 CD4^+^ T cell (213 TPM), naïve CD4^+^ T cell (201 TPM), naïve CD8^+^ T cell (190 TPM), TH17 CD4^+^ T cell (188 TPM), TH1 CD4^+^ T cell (187 TPM), naive TREG CD4^+^ T cell (175 TPM), TH1/17 CD4^+^ T cell (133 TPM), CD4^+^ T cell naïve [activated] (126 TPM), activated CD8^+^ T cell naïve (96 TPM), NK cell CD56 dim CD16^+^ (94 TPM), B cell naïve (80 TPM), monocyte non-classical (52 TPM), monocyte classical (27 TPM) (p=<0.001). Of the all-cell types, T-follicular helper CD4^+^ T cells has shown highest correlation with naïve CD8^+^ T cell (log2 fold change or lfc is 1.33), NK cell CD56 dimCD16^+^ (lfc is 1.49), B cell naïve (lfc is 1.58), Monocyte non-classical (lfc is 1.95), Monocyte classical (lfc is 2.56) (p=<0.001) (Fig. 6**A-C**).

For *PLCG2*, the highest expression was seen in naïve B cells (199 TPM), NK cell CD56dim CD16^+^ (103 TPM), Monocyte non-classical (86 TPM) and Monocyte classical (41 TPM) (p=<0.001). Interestingly, the expression status of *PLCG2* gene in naïve B cells is highly correlated Monocyte classical (lfc is 1.6), T-follicular helper CD4^+^ cells (lfc is 7.27), naive CD8^+^ T cell (lfc is 7.43), naive CD4^+^ T cell (lfc is 7.85), memory TREGs CD4^+^ T cell (lfc is 7.74), TH1 CD4^+^ T cell (lfc is 8.85), activated naive CD8^+^ T cell (lfc is 8.31), naive TREG CD4^+^ T cell (lfc is 9.11), TH17 CD4^+^ T cell (lfc is 8.98), TH1/17 CD4^+^ T cell (lfc is 9.46), TH2 CD4^+^ T cell (lfc is 9.61) and naive [activated] CD4^+^ T cell (lfc is 9.07) (p=<0.001).

In case of *ZEB2,* non-classical monocyte (TPM 106), NK cell CD56dim CD16^+^ (TPM 89), classical monocyte (TPM 84) cells showed the higher expression over naive B cell (TPM 32), TH1 CD4^+^ T cell (TPM 7) and activated naive CD8^+^  T cell (TPM 4) (p=<0.001). The gene expression status of non-classical monocytes is highly correlated with Naïve T cell (lfc is 8.31) and naive TREG CD4^+^ T cells (lfc is 8.47) (p=<0.001).

#### 4.5.1 Drug-gene interaction analysis

Druggability analysis on the three filtered DEGs, i.e., *JAK3, PLCG2, and ZEB2 was done utilizing the DGidb webserver*. Our findings suggest that *JAK3* and *PLCG2* genes have the potential to act as therapeutic targets owing to their drug interaction score of > 0.05. Both of them can be effectively inhibited by Ruxolitinib and Ibrutinib molecules (Table 5). Whereas, no drug targeting *ZEB2* gene was found in our analysis.

**Table 5:** Drug-Gene interaction on DEGs.

**Gene**	**Drug**	**Interaction Type & Directionality**	**Sources**	**Query Score**	**Interaction Score**
*JAK3*	RUXOLITINIB	inhibitor	DTC JAX-CKB GuideToPharmacology	1.83	0.51
	IBRUTINIB	inhibitor)=	GuideToPharmacology	0.38	0.05
*PLCG2*	IBRUTINIB	n/a	CGI DoCM	1.14	8.32

## 5 Discussion

Over the recent decades, systems biology approaches have been widely used to study the gene expression datasets available in open resource databases like GEO and they have identified numerous disease genes and molecules (Sahly et al., 2021, Mujalli et al., 2020). An integrated bioinformatics method is used in this context to identify relevant genes and pathways associated with FH. With help of R Affy and Limma packages, we identified 1363 DEGs for the E-GEOD-13985 dataset and 1266 DEGs for E-GEOD-6088. The comparison of DEGs from both datasets using VENN plot analysis has detected 115 shared genes in the blood expression profile of FH patients.

The GO database is a comprehensive biological resource for understanding protein function. Each gene or protein is annotated against GO terms, falling into distinct ontologies like MF, BP, and CC (du Plessis et al., 2011). The top key enrichment terms enriched for the 115 shared FH genes are the regulation of canonical Wnt signaling pathway under the biological processing category, and mucin type O-glycan biosynthesis pathway (GO:0006488) under the KEGG pathways category. The canonical Wnt signalling, which determines cell proliferation and tissue homeostasis, is known to be modulated by cholesterol through Dishevelled (Dvl) protein. Dysregulation of Wnt signalling in FH patients supports that hyper cholesterololemia reduces the risk of cancer development (Sheng et al., 2014). Moreover, dysregulation of the mucin type O-glycan pathway may negatively impact the O-glycosylation of class A repeats in LDLR, whose stable expression is essential for LDL absorption into cells (Pedersen et al., 2014).

The continuous deposition of cholesterol and other lipids in the blood vessel wall promotes local hyperplasia, cytokine secretions, macrophage invasions and macrophage foam cell formation, which are the eventual underlying cause of early onset of atherosclerosis and cardiovascular diseases (Falk, 2006, Collaboration EASFHS et al., 2016, Heusch et al., 2014). However, rather than single gene actions, the chronic disease like atherosclerosis involves the action of perturbed protein networks, Therefore, based on the DEGs identified in FH patients, we initially constructed protein–protein networks and further decomposed it into a functional cluster. In this context, we extended the protein network cluster with 9 FH candidate genes (*APOA2, APOB, GHR, LDLR, LDLRAP1, ABCA1, PCSK9, SMARCA4,* and *EPHX2*) and performed their GO annotations.

Network clusters are characterized by extensive connectivity between a set of genes, and GO annotations provide their biological interpretation (Zhong and Xie, 2007). The top significant GO terms in the biological processes category were the regulation of intracellular signal transduction and positive regulation of transcription, DNA-templated. The dysfunction of lipid homeostasis, PI3K/AKT signaling transduction pathways, monocyte chemotaxis, macrophage migration, and neovascularization are some of the atherosclerotic plaque formation characteristics (Li et al., 2018, Zhao et al., 2021). The accumulation of LDL particles leads to the formation and deposition of fibrous plaques in the subendothelial space, the eventual narrowing of arterial diameter leads to heart ischemia and myocardial infarction (35). In KEGG pathways category, thyroid hormone signaling pathway was the key enrichment term followed by ErbB signaling pathway. Since thyroid hormones are known to influence lipid homeostasis in the liver, hypothyroidism can cause hypercholesterolemia, which is commonly observed in patients with hypothyroidism (Delitala et al., 2017). Hypercholesterolemia in hypothyroidism leads to simultaneous diminishing control by triiodothyronine (T3) of SREBP-2 protein, which regulate the synthesis of cholesterol by modifying the HMG-CoA enzyme activity (Duntas and Brenta, 2018). Cholesterol levels are known to influence EGFR signaling processes by negatively affecting the receptor function and trafficking (Pike and Casey, 2002). Moreover, EGFR inhibition is shown to be useful for the treatment of hypercholesterolemia in high-fat-diet-fed Mitogen-inducible gene 6 (Mig-6) Mig-6^d/d^ mice (Lee et al., 2014). GO results have shown that 5 (*CSNK1A1, JAK3, PLCG2, RALA, ZEB2*) network cluster genes were commonly enriched against all annotation terms. Then open target platform-based correlations between the target gene and disease phenotypes revealed that *ZEB2, JAK3* and *PLCG2* genes are highly associated with cardiovascular and immunology related phenotypes.

The *JAK3* is a tyrosine kinase protein, whose critical role in the cytokine receptor signal transduction pathway is very important for T-lymphocyte differentiation and function (Murray, 2007). A defective JAK3 signalling pathway is important for a multitude of immune-associated disorders, includes atherosclerotic process. Our immune cell restriction analysis findings show that *JAK3* expression is highly correlated with T-follicular helper CD4^+^T cells, which stimulate atherosclerosis and additional CVDs (Methe et al., 2005). *JAK3* has been an ideal molecular target for several immunomodulators (García-Bermúdez et al., 2015, García-Bermúdez et al., 2015). Ruxolitinib is a dual JAK1 and JAK2 inhibitor with biological IC50s in the single digit nanomolar range for both kinases. A sixfold selectivity over Tyk2 and approximately 130-fold selectivity over JAK3 is represented within the JAK family members (Wu et al., 2019). Ibrutinib (PCI-32765) is a strong Brutons tyrosine kinase (Btk) inhibitor with an IC50 of 0.5 nM in cell-free tests. It is shown to act as a potent inhibitor to Bmx, CSK, FGR, BRK, and HCK, but less potent to EGFR, Yes, ErbB2, JAK3, and other kinases (van Vollenhoven et al., 2015).

The second gene *PLCG2*, drives the hydrolysis of membrane phospholipids to secondary messengers IP3 and diacylglycerol using calcium as a cofactor. *PLCG2* variants are reported to contribute to autoinflammation and immune dysregulation (Sims et al., 2017). Our immune restriction analysis findings have confirmed the elevated expression of *PLCG2* in naïve B cells, NK cells, and monocytes. The third gene *ZEB2* belongs to the Zfh1 family of zinc finger/homeodomain proteins, which act as DNA binding transcriptional inhibitors (Bar Yaacov et al., 2018). The activity of *ZEB2* is strongly related to dyslipidemia, metabolic changes, and CD8 + T cell alterations seen in atherosclerotic plaques (Fernandez, et al., 2020). Our immune cell restriction analysis results show that monocytes show higher expression of ZEB2 than native B cells or TH1 CD4 + T cell. In the hematopoietic system, Zeb2 together with Tbx21 (Tbet) promotes natural killer (NK) cell maturation and CD8 + T cells, and inactivation of *ZEB2* dysregulates hematopoiesis with neutrophilia and monocyte loss, which validates our findings (Li et al., 2017).

Even though the current study used a thorough bioinformatic analysis, some technical limitations or weaknesses could not be ruled out. Owing to the small amount of data, our results could not be generalized to a larger number of FH patients. The accuracy of the analytical findings can be improved by enlarging the samples. Furthermore, while it can be described to certain extent that hub genes are strongly involved in FH pathogenesis and may potentially serve as key biomarkers for therapeutic molecules, future research work on animal models or cell line models is required to validate our findings.

## 6 Conclusion

This study has detected the expression differences of 115 genes in the FH samples compared to the controls. Functional ontology findings have linked these genes to intracellular lipoprotein metabolism, immune responses, cell adhesion molecules, canonical Wnt signaling, mucin type O-glycan biosynthesis pathways in FH patients. The expanded protein interaction network construction,GO annotations, and open target analysis has revealed *ZEB2, JAK3* and *PLCG2* genes as the key hub genes, which connects cardiovascular and immune response related phenotypes. These findings not only offer a hypothetical basis for better understanding immune dysregulation mechanisms underlying the atherosclerosis among FH patients, but also open the path for the development of therapeutic targets to reduce the cardiovascular disease burden in FH patients.

## Ethical approval

This study does not require any ethical approval as the publicly available gene expression datasets have been used.

## Data availability statement

The article includes all datasets studied for this investigation.

## CRediT authorship contribution statement

**Zuhier Awan:** Conceptualization, Data curation, Funding acquisition, Methodology, Project administration, Validation, Writing – original draft. **Nuha Al-Rayes:** Methodology. **Zeenath Khan:** . **Majid Al Mansouri:** Data curation, Validation. **Abdulhadi Ibrahim H. Bima:** Validation. **Haifa Almukadi:** Validation. **Hussam Ibrahim Kutbi:** Methodology, Validation. **Preetha Jayasheela Shetty:** Methodology, Validation. **Noor Ahmad Shaik:** Conceptualization, Validation, Writing – original draft. **Babajan Banaganapalli:** Conceptualization, Data curation, Formal analysis, Methodology, Software, Validation, Visualization, Writing – original draft.

## Declaration of Competing Interest

The authors declare that they have no known competing financial interests or personal relationships that could have appeared to influence the work reported in this paper.

## References

[b0005] Awan Z.A., Rashidi O.M., Al-Shehri B.A., Jamil K., Elango R., Al-Aama J.Y., Hegele R.A., Banaganapalli B., Shaik N.A. (2021). Saudi Familial Hypercholesterolemia Patients With Rare LDLR Stop Gain Variant Showed Variable Clinical Phenotype and Resistance to Multiple Drug Regimen. Front Med (Lausanne)..

[b0010] Alhabib K.F., Al-Rasadi K., Almigbal T.H., Batais M.A., Al-Zakwani I., Al-Allaf F.A., Al-Waili K., Zadjali F., Alghamdi M., Alnouri F., Awan Z., Kinsara A.J., AlQudaimi A., Almahmeed W., Sabbour H., Traina M., Atallah B., Al-Jarallah M., AlSarraf A., AlSayed N., Amin H., Altaradi H., Cheng X. (2021). Familial Hypercholesterolemia in the Arabian Gulf Region: Clinical results of the Gulf FH Registry. PLoS ONE.

[b0015] Fantus D., Awan Z., Seidah N.G., Genest J. (2013). Aortic calcification: Novel insights from familial hypercholesterolemia and potential role for the low-density lipoprotein receptor. Atherosclerosis..

[b0020] Villa G., Wong B., Kutikova L., Ray K.K., Mata P., Bruckert E. (2017). Prediction of cardiovascular risk in patients with familial hypercholesterolaemia. Eur. Heart J. Qual. Care Clin. Outcomes..

[b0025] Berberich A.J., Hegele R.A. (2019). The complex molecular genetics of familial hypercholesterolaemia. Nat. Rev. Cardiol..

[b0030] Andersen L.H., Miserez A.R., Ahmad Z., Andersen R.L. (2016). Familial defective apolipoprotein B-100: A review. J. Clin. Lipidol..

[b0035] Abifadel M., Varret M., Rabès J.-P., Allard D., Ouguerram K., Devillers M., Cruaud C., Benjannet S., Wickham L., Erlich D., Derré A., Villéger L., Farnier M., Beucler I., Bruckert E., Chambaz J., Chanu B., Lecerf J.-M., Luc G., Moulin P., Weissenbach J., Prat A., Krempf M., Junien C., Seidah N.G., Boileau C. (2003). Mutations in PCSK9 cause autosomal dominant hypercholesterolemia. Nat. Genet..

[b0040] Warden B.A., Fazio S., Shapiro M.D. ((MA)2000.).

[b0045] Raal F.J., Santos R.D. (2012). Homozygous familial hypercholesterolemia: current perspectives on diagnosis and treatment. Atherosclerosis..

[b0050] Paththinige C.S., Sirisena N.D., Dissanayake V. (2017). Genetic determinants of inherited susceptibility to hypercholesterolemia - a comprehensive literature review. Lipids Health Dis..

[b0055] Brænne I., Reiz B., Medack A., Kleinecke M., Fischer M., Tuna S., Hengstenberg C., Deloukas P., Erdmann J., Schunkert H. (2014). Whole-exome sequencing in an extended family with myocardial infarction unmasks familial hypercholesterolemia. BMC Cardiovasc. Disorders..

[b0060] Wu W.-F., Sun L.-Y., Pan X.-D., Yang S.-W., Wang L.-Y., Veitia R.A. (2014). Use of targeted exome sequencing in genetic diagnosis of Chinese familial hypercholesterolemia. PLoS ONE.

[b0065] Marks D., Thorogood M., Neil H.A.W., Humphries S.E. (2003). A review on the diagnosis, natural history, and treatment of familial hypercholesterolaemia. Atherosclerosis..

[b0070] Li X., Lalić J., Baeza-Centurion P., Dhar R., Lehner B. (2019). Changes in gene expression predictably shift and switch genetic interactions. Nat. Commun..

[b0075] Soutar A.K., Naoumova R.P. (2007). Mechanisms of disease: genetic causes of familial hypercholesterolemia. Nat. Clin. Pract. Cardiovasc. Med..

[b0080] Qin W., Qian S.X., Cai X.H., Lu X.Z., Chao H.Y. (2021). Analysis of the Differential Expression of circRNA in Acute Myeloid Leukemia by GEO Database. Zhongguo shi yan xue ye xue za zhi..

[b0085] Sabir JSM, El Omri A, Banaganapalli B, et al. Dissecting the Role of NF-κb Protein Family and Its Regulators in Rheumatoid Arthritis Using Weighted Gene Co-Expression Network. Front Genet. 2019;10:1163. 10.3389/fgene.2019.01163 PMC6879671 31824568

[b0090] Sabir JSM, El Omri A, Banaganapalli B, et al. Unraveling the role of salt-sensitivity genes in obesity with integrated network biology and co-expression analysis. *PLoS One.* 2020;15(2):e0228400. 10.1371/journal.pone.0228400 PMC7004317 32027667

[b0095] Banaganapalli B., Al-Rayes N., Awan Z.A., Alsulaimany F.A., Alamri A.S., Elango R., Malik M.Z., Shaik N.A. (2021). Multilevel systems biology analysis of lung transcriptomics data identifies key miRNAs and potential miRNA target genes for SARS-CoV-2 infection. Comput. Biol. Med..

[b0100] Banaganapalli B., Mansour H., Mohammed A., Alharthi A.M., Aljuaid N.M., Nasser K.K., Ahmad A., Saadah O.I., Al-Aama J.Y., Elango R., Shaik N.A. (2020). Exploring celiac disease candidate pathways by global gene expression profiling and gene network cluster analysis. Sci. Rep..

[b0105] Huang S. (1999). Gene expression profiling, genetic networks, and cellular states: an integrating concept for tumorigenesis and drug discovery. J. Mol. Med. (Berlin, Germany)..

[b0110] Craven K.E., Gokmen-Polar Y., Badve S.S. (2021). CIBERSORT analysis of TCGA and METABRIC identifies subgroups with better outcomes in triple negative breast cancer. Sci Rep..

[b0115] Xie Z., Bailey A., Kuleshov M.V., Clarke D.J.B., Evangelista J.E., Jenkins S.L., Lachmann A., Wojciechowicz M.L., Kropiwnicki E., Jagodnik K.M., Jeon M., Ma'ayan A. (2021). Gene Set Knowledge Discovery with Enrichr. Current protocols..

[b0120] Athar A., Füllgrabe A., George N., Iqbal H., Huerta L., Ali A., Snow C., Fonseca N.A., Petryszak R., Papatheodorou I., Sarkans U., Brazma A. (2019). ArrayExpress update - from bulk to single-cell expression data. Nucleic Acids Res..

[b0125] Režen T.C.A., Brecelj N., Rozman D., Keber I., Fon T.K. (2019). Atherosclerotic markers in human blood - a study in patients with familial hypercholesterolemia. GEO..

[b0130] Mosig S., Rennert K., Büttner P., Krause S., Lütjohann D., Soufi M., Heller R., Funke H. (2008). Monocytes of patients with familial hypercholesterolemia show alterations in cholesterol metabolism. BMC Med. Genom..

[b0135] Irizarry R.A., Hobbs B., Collin F. (2003). Exploration, normalization, and summaries of high density oligonucleotide array probe level data. Biostatistics..

[b0140] Gautier L., Cope L., Bolstad B.M., Irizarry R.A. (2004). affy–analysis of Affymetrix GeneChip data at the probe level. Bioinformatics.

[b0145] Ritchie M.E., Silver J., Oshlack A., Holmes M., Diyagama D., Holloway A., Smyth G.K. (2007). A comparison of background correction methods for two-colour microarrays. Bioinformatics (Oxford, England)..

[b0150] Gentleman R.C., Carey V.J., Bates D.M. (2004). Bioconductor: open software development for computational biology and bioinformatics. Genome Biol..

[b0155] Szklarczyk D., Morris J.H., Cook H., Kuhn M., Wyder S., Simonovic M., Santos A., Doncheva N.T., Roth A., Bork P., Jensen L.J., von Mering C. (2017). The STRING database in 2017: quality-controlled protein-protein association networks, made broadly accessible. Nucleic Acids Res..

[b0160] Clarke D.J.B., Jeon M., Stein D.J., Moiseyev N., Kropiwnicki E., Dai C., Xie Z., Wojciechowicz M.L., Litz S., Hom J., Evangelista J.E., Goldman L., Zhang S., Yoon C., Ahamed T., Bhuiyan S., Cheng M., Karam J., Jagodnik K.M., Shu I., Lachmann A., Ayling S., Jenkins S.L., Ma'ayan A. (2021). Appyters: Turning Jupyter Notebooks into data-driven web apps. Patterns (New York, NY)..

[b0165] Shannon P., Markiel A., Ozier O., Baliga N.S., Wang J.T., Ramage D., Amin N., Schwikowski B., Ideker T. (2003). Cytoscape: a software environment for integrated models of biomolecular interaction networks. Genome Res..

[b0170] Bader G.D., Hogue C.W. (2003). An automated method for finding molecular complexes in large protein interaction networks. BMC Bioinf..

[b0175] Supek F., Bošnjak M., Škunca N., Šmuc T., Gibas C. (2011). REVIGO summarizes and visualizes long lists of gene ontology terms. PLoS ONE.

[b0180] Cotto K.C., Wagner A.H., Feng Y.-Y., Kiwala S., Coffman A.C., Spies G., Wollam A., Spies N.C., Griffith O.L., Griffith M. (2018). DGIdb 3.0: a redesign and expansion of the drug-gene interaction database. Nucleic Acids Res..

[b0185] Sahly N.N., Banaganapalli B., Sahly A.N., Aligiraigri A.H., Nasser K.K., Shinawi T., Mohammed A., Alamri A.S., Bondagji N., Elango R., Shaik N.A. (2021). Molecular differential analysis of uterine leiomyomas and leiomyosarcomas through weighted gene network and pathway tracing approaches. Syst. Biol. Reprod. Med..

[b0190] Mujalli A., Banaganapalli B., Alrayes N.M., Shaik N.A., Elango R., Al-Aama J.Y. (2020). Myocardial infarction biomarker discovery with integrated gene expression, pathways and biological networks analysis. Genomics.

[b0195] du Plessis L., Skunca N., Dessimoz C. (2011). The what, where, how and why of gene ontology–a primer for bioinformaticians. Brief Bioinform..

[b0200] Sheng R., Kim H., Lee H., Xin Y., Chen Y., Tian W., Cui Y., Choi J.-C., Doh J., Han J.-K., Cho W. (2014). Cholesterol selectively activates canonical Wnt signalling over non-canonical Wnt signalling. Nat. Commun..

[b0205] Pedersen N.B., Wang S., Narimatsu Y., Yang Z., Halim A., Schjoldager K.-B., Madsen T.D., Seidah N.G., Bennett E.P., Levery S.B., Clausen H. (2014). Low Density Lipoprotein Receptor Class A Repeats Are O-Glycosylated in Linker Regions*. J. Biol. Chem..

[b0210] Falk E. (2006). Pathogenesis of atherosclerosis. J Am Coll Cardiol..

[b0215] Collaboration EASFHS, Vallejo-Vaz AJ, Akram A, et al. Pooling and expanding registries of familial hypercholesterolaemia to assess gaps in care and improve disease management and outcomes: Rationale and design of the global EAS Familial Hypercholesterolaemia Studies Collaboration. *Atheroscler Suppl.* 2016;22:1-32. 10.1016/j.atherosclerosissup.2016.10.001 27939304

[b0220] Heusch G., Libby P., Gersh B., Yellon D., Böhm M., Lopaschuk G., Opie L. (2014). Cardiovascular remodelling in coronary artery disease and heart failure. Lancet.

[b0225] Zhong S., Xie D. (2007). Gene Ontology analysis in multiple gene clusters under multiple hypothesis testing framework. Artif. Intell. Med..

[b0230] Li W., Huang H., Li L., Wang L.i., Li Y., Wang Y., Guo S., Li L., Wang D., He Y., Chen L. (2018). The Pathogenesis of Atherosclerosis Based on Human Signaling Networks and Stem Cell Expression Data. Int. J. Biol. Sci..

[b0235] Zhao Y., Qian Y., Sun Z., Shen X., Cai Y., Li L., Wang Z. (2021). Role of PI3K in the Progression and Regression of Atherosclerosis. Front. Pharmacol..

[b0240] Delitala A.P., Delitala G., Sioni P., Fanciulli G. (2017). Thyroid hormone analogs for the treatment of dyslipidemia: past, present, and future. Curr. Med. Res. Opin..

[b0245] Duntas L.H., Brenta G. (2018). A Renewed Focus on the Association Between Thyroid Hormones and Lipid Metabolism. Front. Endocrinol..

[b0250] Pike L.J., Casey L. (2002). Cholesterol levels modulate EGF receptor-mediated signaling by altering receptor function and trafficking. Biochemistry.

[b0255] Lee J.C., Park B.K., Choung S., Kim J.M., Joung K.H., Lee J.H., Kim K.S., Kim H.J., Jeong J.-W., Rhee S.D., Ku B.J., He B. (2014). Amelioration of hypercholesterolemia by an EGFR tyrosine kinase inhibitor in mice with liver-specific knockout of Mig-6. PLoS ONE.

[b0260] Murray P.J. (2007). The JAK-STAT signaling pathway: input and output integration. J. Immunol..

[b0265] Methe H., Brunner S., Wiegand D., Nabauer M., Koglin J., Edelman E.R. (2005). Enhanced T-helper-1 lymphocyte activation patterns in acute coronary syndromes. J. Am. Coll. Cardiol..

[b0270] García-Bermúdez M., López-Mejías R., Genre F. (2015). Lack of Association between <i>JAK3</i> Gene Polymorphisms and Cardiovascular Disease in Spanish Patients with Rheumatoid Arthritis. Biomed Res. Int..

[b0275] García-Bermúdez M., López-Mejías R., Genre F., Castañeda S., Corrales A., Llorca J., González-Juanatey C., Ubilla B., Miranda-Filloy J.A., Pina T., Gómez-Vaquero C., Rodríguez-Rodríguez L., Fernández-Gutiérrez B., Balsa A., Pascual-Salcedo D., López-Longo F.J., Carreira P., Blanco R., Martín J., González-Gay M.A. (2015). Lack of association between JAK3 gene polymorphisms and cardiovascular disease in Spanish patients with rheumatoid arthritis. Biomed. Res Int..

[b0280] Wu C.-P., Hsu S.-C., Chen Z.-.-S., Yang D.-.-H. (2019).

[b0285] van Vollenhoven R.F., Hochberg M.C., Silman A.J., Smolen J.S., Weinblatt M.E., Weisman M.H. (2015). Rheumatology.

[b0290] Sims R., van der Lee S.J., Naj A.C. (2017). Rare coding variants in PLCG2, ABI3, and TREM2 implicate microglial-mediated innate immunity in Alzheimer's disease. Nat. Genet..

[b0295] Bar Yaacov R., Eshel R., Farhi E., Shemuluvich F., Kaplan T., Birnbaum R.Y. (2018). Functional characterization of the ZEB2 regulatory landscape. Hum. Mol. Genet..

[b0300] Fernandez D, Fernandez NF, Rahman a, et al. Abstract 258: ZEB2 Regulates Activation and Exhaustion Programming of CD8<sup>+</sup> T Cells in Atherosclerosis. *Arteriosclerosis, Thrombosis, and Vascular Biology.* 2020;40(Suppl_1):A258-A258.

[b0305] Li J., Riedt T., Goossens S., Carrillo García C., Szczepanski S., Brandes M., Pieters T., Dobrosch L., Gütgemann I., Farla N., Radaelli E., Hulpiau P., Mallela N., Fröhlich H., La Starza R., Matteucci C., Chen T., Brossart P., Mecucci C., Huylebroeck D., Haigh J.J., Janzen V. (2017). The EMT transcription factor Zeb2 controls adult murine hematopoietic differentiation by regulating cytokine signaling. Blood.

